# Positional Differences in Youth Water Polo Players: Cognitive Functions, Specific Swimming Capacities and Anthropometric Characteristics

**DOI:** 10.3390/jfmk10020151

**Published:** 2025-04-28

**Authors:** Neven Kovačević, Frane Mihanović, Linda Lušić Kalcina, Tatjana Matijaš, Tea Galić

**Affiliations:** 1 Faculty of Kinesiology, University of Split, 21000 Split, Croatia; nevenkovacevic@hotmail.com; 2 Croatian Water Polo Federation, 10000 Zagreb, Croatia; 3 University Department of Health Sciences, University of Split, 21000 Split, Croatia; frane.mihanovic@ozs.unist.hr (F.M.); tmatijas@ozs.unist.hr (T.M.); 4 Department of Neuroscience, University of Split School of Medicine, 21000 Split, Croatia; linda.lusic.kalcina@mefst.hr; 5 Department of Prosthodontics, Study of Dental Medicine, University of Split School of Medicine, 21000 Split, Croatia

**Keywords:** water polo, cognitive functions, psychomotor ability, inhibition

## Abstract

**Objectives:** Water polo players ought to possess various physical capacities and well-developed cognitive functions that reflect the requirements of their specific playing position. Therefore, the objective of this study was to compare the cognitive performance, anthropometric characteristics and specific swimming capacities of youth water polo players in different playing positions. **Methods:** The present cross-sectional study involved 106 youth water polo players. The subjects were recruited as part of a project for talent identification and selection for the Croatian National Water Polo Team. Testing included anthropometric measurements, specific swimming capacities and cognitive performance (Stroop test). **Results:** Among the 106 youth water polo players, there were 15 goalkeepers (14.2%), 21 center-defenders (19.8%), 17 center-forwards (16.0%), 34 drivers (32.1%) and 19 wings (17.9%), with the mean age of 14.14 ± 0.38 years. The wings performed faster than center-forwards in both StroopOff time (wings: 57.14 ± 10.04 s vs. center-forwards: 67.03 ± 9.72 s, *p* = 0.016) and StroopOn time (wings: 66.18 ± 15.86 s vs. center-forwards: 80.24 ± 15.64 s, *p* = 0.019). **Conclusions:** In conclusion, this study demonstrated significant differences between different playing positions in youth water polo players, specifically between center-forwards and wings. They performed faster than center-forwards in all tested variables of the Stroop test, measures of psychomotor ability, response inhibition and motor speed, as well as in specific swimming capacities measured in the 50 m crawl and the 400 m crawl. The results of this study provide a valuable foundation for establishing developmental recommendations for different playing positions, aimed at improving player’s performance. These recommendations should take into account anthropometric characteristics, specific functional swimming capacities and cognitive functions that influence players’ game intelligence, which can be enhanced through properly designed training programs.

## 1. Introduction

### 1.1. Cognitive Functions

Over the last two decades, there has been a growing interest in sports science, including perceptual–cognitive processes in sports, talent identification and performance success [1,2,3,4,5,6,7,8]. Nowadays, it has become widely accepted that for success in sports, an athlete should have a combination of extraordinary physiological capacities (aerobic and anaerobic capacity), specific technical and tactical skills and psychological and emotional characteristics (adaptation, emotional control and self-efficacy), as well as cognitive functions related to game strategy, such as perception, executive functions, attention, anticipation and decision-making [9,10,11,12,13]. The preparation program involves physical and cognitive components particular to a sport discipline, the level of performance, and the role of the player within a team [3,14,15,16,17]. Since all players obtain adequate physical and technical abilities, cognitive skills make a sharp distinction between players, positioning them in completely different athletic dimensions [18]. The ability to make prompt decisions is a substantial expectation in the majority of sports, especially in the case of fast and dynamic team sports like water polo. Such skills make a player capable to choose the most appropriate action for the given situation during the game [13,18,19]. The results of the meta-analysis performed by Kalen et al. [3] reported that high-level players demonstrated better cognitive decision-making skills than their low-level peers, indicating that these skills might be an important feature for athletic performance and success. Additionally, previous research by Kovačević et al. [20] showed that youth water polo players selected in the national team outperformed their peers in the results of the Stroop test who did not reach that level of water polo performance. Similarly, Trecorci et al. [21] showed a large positive correlation (*r* = 0.45, d-value = 1.01) of the cumulated score summarizing cognitive functions with the cumulated score summarizing sport-specific physical performance, suggesting that volleyball athletes with superior basic cognitive functions present better sport-specific physical performance. Vestberg et al. [8] also showed that high division soccer players outperformed low division soccer players, in general, in terms of executive functions, which was also correlated with the numbers of goals and assists the players had scored two seasons later [3,8,20,21,22,23]. The results of previous research have also indicated that athletes perform better on selected cognitive tasks compared to the general population of the same age [18,19,24,25].

Similar to sport-specific requirements for physical and technical–tactical preparation, cognitive skills also vary for each type of sport, for different situations within a particular sport as well as for different playing positions [26,27,28]. Therefore, it can be assumed that in water polo, for particular positions of players on a field, cognitive features such as concentration (enabling the player to make the right decisions faster and to make fewer mistakes); peripheral vision (enabling the player to notice teammates and the opponents on the sides); decision-making, which enables the selection of the appropriate response in the specific playing environment; short-term memory (enabling the player to remember the position of teammates and the opponents on the field); and reaction time (due to the high pace of the game, quick throws, etc.) will play an essential role in athletic performance [17,26,29,30].

### 1.2. Water Polo

Among team sports, water polo is definitely a high-demanding one as it is characterized by organized physical contact among players. Water polo is a dynamic and strategic sport that not only requires physical strength and stamina but also engages a variety of cognitive functions to excel [17,31,32,33]. The combination of swimming, ball handling, and tactics in a water polo game demands a high level of mental processing, from decision-making to communication among teammates [34,35,36]. Anthropometric characteristics and swimming capacity play important roles related to the general performance level of a youth water polo player, as previously pointed out by various authors [17,20,31,37,38,39,40,41]. Although fewer studies have been published in the field of water polo compared to other sports, such as swimming, handball or soccer, position-specific differences in the anthropometric characteristics of the players were reported [37,39,40,41]. These studies can provide a good basis for developing position-specific training programs by taking the anthropometric and body composition characteristics of the players into account. Accordingly, to develop position-specific training programs, players’ cognitive functions and mental strength should also be considered. Moreover, it has been proven that in addition to the physiological and biomechanical demands, technical, tactical and water polo-specific skills are important factors for successful participation at the elite level [20,33,42,43]. Players must make quick decisions in high-pressure situations. For example, when attacking or defending, they need to recognize opportunities (e.g., open teammates and a chance to shoot) and threats (e.g., defensive pressure or potential counterattacks) and respond swiftly. Reaction time is crucial, particularly when intercepting passes, defending shots, or responding to the movements of opposing players [44,45]. According to former studies, only a combination of performance characteristics can determine a player’s performance level [17,28,36,46,47,48].

### 1.3. Playing Positions in Water Polo

A number of different playing positions in water polo show different game demands. Goalkeepers are required to constantly tread water between the goals and block shots made toward the goal. As the goalkeeper is the last line of defense and the first line of offense, a goalkeeper is expected to be courageous, agile, flexible and psychologically stable with a high level of concentration, reacting quickly to changing situations. They must have quick reflexes, excellent hand–eye coordination and the ability to read the opponent’s offensive movements [27,38,44,49]. A center-forward plays between defenders, where he has to fight for the position and create a throw-in for himself. Therefore, he is expected to have the physical strength and high body mass required to engage in physical struggles with his opponent to hold key positions in the pool [28,34,50]. From a cognitive perspective, the center-forward is expected to have a wide range of external attention; the ability of multi-switching, positioning, reading the defense and decision-making under pressure; and a high level of working memory [28,33,38]. Wings are required to do large amounts of swimming up and down the sides of the pool to create scoring opportunities for their team and to prevent those of their opponents. The main objective of the wing position is to swim down to the 2 m line and be open to receive a pass. Wings are in a great position to make entry passes to the hole set, which can often result in an exclusion or kick out. By passing the ball to the wings, the entire offense is able to move closer to the goal for better scoring chances [32,33,37,40,44]. Therefore, wings need to have the greatest speed, agility and tactical awareness. Drivers are typically fast players that drive through the offense to create movement and goal scoring opportunities. The driver is usually the shooter in the team, playing the role of a defender as well, which requires multi-tasking and a high level of strength and fitness, similar as in handball [27,44,51]. The center-defender has the task of organizing actions and passing the ball effectively to teammates. This player needs strong defensive instincts and the ability to predict and block passes and shots. He is expected to make quick and accurate decisions, anticipate plays and demonstrate quick thinking [27,44,51].

Previous studies in water polo identified differences between specific playing positions, mostly in anthropometric characteristics, functional capacities or physical fitness profiles [37,40,52,53]. To optimize team performance, players in different positions along with different physical characteristics need to possess well-developed cognitive functions that reflect the requirements of their playing position [53,54,55]. The importance of cognitive functions in youth water polo players has been demonstrated in our previous study, where players selected to the youth national water polo team demonstrated better cognitive functions (psychomotor ability, inhibition, motor speed and cognitive flexibility) than non-selected players of the same age [20].

To the best of our knowledge, no studies have examined cognitive functions in water polo players according to their playing positions. Therefore, the objective of this study was to compare the cognitive performance of youth water polo players playing in different positions, along with the anthropometric characteristics and specific swimming capacities. Considering previously described specific demands of each playing position in water polo, the null hypothesis of our study was that wings will outperform players in other playing positions, specifically center-forwards, in cognitive functions. In addition, center-forwards will dominate through their anthropometric characteristics and show lower specific swimming capacities in comparison to other playing positions.

## 2. Materials and Methods

### 2.1. Participants

The present study involved 106 youth water polo players attending Croatian Water Polo Federation training camps for male water polo players under 14 years old (U14). The subjects recruited were part of a project for talent identification and selection to the Croatian National Water Polo Team. All participants had over two years of competitive water polo experience, with an average of five training sessions per week. At the time of data collection, their teams were playing at the top level of their respective age group (U14). Participants did not report any behavioral, learning or medical condition that might influence their cognitive function. Their physical fitness and educational level were comparable. There were 15 goalkeepers (14.2%), 21 center-defenders (19.8%), 17 center-forwards (16.0%), 34 drivers (32.1%) and 19 wings (17.9%), with an average age of 14.14 ± 0.38 years. This study was performed in accordance with the Declaration of Helsinki and approved by the Ethical Committee of the University of Split School of Medicine, Split, Croatia (No.: 2181-198-03-04-19-0053). All players and their parents/legal guardians received the detailed description of the study before providing their written informed consent.

### 2.2. Study Protocol

This study was performed from season 2019/2020 until 2023/2024 following the same protocol as in our previous studies [20,41]. The inclusion criteria were as follows: attendance in the Croatian Water Polo Federation camps, being active in water polo for at least 2 years, having at least 5 training sessions per week and played in the highest league for their age group. The exclusion criteria were color blindness and the inability to fulfill the study protocol. At the end of the season, when national championships for the U14 age category were finished, players from across the nation were invited to a 4-day training camp, specific for the U14 age category. On the first day, anthropometric measurements (body mass and body height) were collected, and subjects wore only swimming trunks. Swimming tests were performed in following order: first day: 400 m front crawls for field players and a 100 m butterfly test for goalkeepers; second day: 25 m front crawls and 25 m of ball dribbling; third day: 50 m front crawls and cognitive testing; and fourth day: 100 m front crawls for field players and a 100 m breaststroke test for goalkeepers. For cognitive evaluation focused on psychomotor ability, inhibition and motor speed the Stroop test with the following specification was conducted: early in the morning right after the breakfast, between 8:00 a.m. and 9:00 a.m. and in the quiet, bright room, while all swimming tests were performed in the morning from 9:00 a.m. and 11:00 a.m. in order to avoid diurnal variations.

### 2.3. Anthropometric Measurements

Anthropometric measurements were carried out according to conventional criteria and measurement procedures. Body mass was measured using a 100 g precision digital scale under standard conditions (wearing only swimming trunks). Height was measured with a 1 mm precision wall ruler. The body mass index (BMI) was calculated as body weight (kg) divided by height squared (m^2^).

### 2.4. Specific Functional Swimming Tests

Specific functional swimming tests included a 25 m front crawl, a 50 m front crawl, a 100 m front crawl, a 400 m front crawl and 25 m of dribbling for field players and a 100 m butterfly test and a 100 m breaststroke test for goalkeepers. The testing was carried out in a 25 m pool; the start was at the signal sound from the water; the players were permitted to push off the wall, but in order for the test to be homogeneous, flip-turns were not allowed. Prior to the testing, the participants completed a 15 min convenient warm-up procedure, consisting of a dry land warm-up and 10–15 min of swimming, using different swimming techniques. The testing was carried out in groups of eight participants. The participants were timed with a hand-held digital stopwatch (Longines, Saint-Imier, Switzerland).

### 2.5. Cognitive Performance

For cognitive functions testing, a touchscreen version of the Stroop test (The EncephalApp_Stroop application, version 15.6.1) was downloaded on tablets. Participants were tested under controlled environmental conditions in groups of 10 in an adequately prepared room for such research, i.e., proper lighting of the room, proper setting of the apparatus, and constant temperature; this ensured that none of the participants were disturbed. Players were asked to perform tasks according to the instructions presented on a computer screen. Throughout the test, two researchers (T.G. and F.M.) were present in the room and assisted participants as needed (e.g., when there were technical problems, interpretation of the task, etc.). During the testing procedure, participants sat at a table, with the dominant limb’s hand open and placed at the table’s edge. Participants were requested to respond as quickly and accurately as possible. The task included trials in two different conditions, i.e., congruent (StroopOff) and incongruent (StroopOn). At the beginning of the test, two training runs were given for each condition. In the easier StroopOff condition, the participants viewed a neutral stimulus, namely hashtag signs (###) that were presented in red, green, or blue, one at a time and had to respond as quickly as possible by touching the matching color of the stimulus to the color displayed at the bottom of the screen. The colors at the bottom of the screen were shown in a randomized order that was not fixed to their respective positions. If the participant made a mistake, such as pressing the wrong color, the run stopped, and he had to restart again. In the StroopOn condition, which is a more challenging part of the test, the participants were instructed to ignore the meaning of the words and to focus on the colors in which the words were printed on the screen. Both conditions were continued until five correct runs were achieved, therefore also indicating the number of mistakes. The specific outcomes of the Stroop test included the total time for five correct runs in the “Off” condition (OffTime), which primarily assessed their psychomotor ability; total time for five correct runs in the “On” condition (OnTime), which is a measure of response inhibition and motor speed; as well as the number of runs needed to complete the five correct “Off” and “On” runs. The test of cognitive processing controlling for psychomotor speed was subtracting the OffTime from the OnTime (OnTime minus OffTime), while OffTime + OnTime showed a composition measure of psychomotor speed and response inhibition [56,57,58,59]. The reliability of the Stroop test was tested using Pearson’s correlation coefficient in test–retest analysis. It was performed in 24 participants, showing correlation coefficients for OffTime *r* = 0.872 (95% CI 0.723–0.944) and OnTime *r* = 0.890 (95% CI 0.760–0.952), respectively. A more detailed description of the test can be found in our previous study [20]. The mean response time was computed for each condition, considering only correct responses.

### 2.6. Statistical Analyses

Statistical analyses were performed using the MedCalc statistical software (MedCalc Software for Windows, version 19.4, MedCalc Software Ltd., Ostend, Belgium). The assumption of homogeneity of the variance was tested using Levene’s test, and the assumption of normality was checked using the Kolgomorov–Smirnov test. One-way ANOVA with the Scheffé post hoc test was used to determine the differences between the variables measured (anthropometric characteristics, specific functional swimming capacities and cognitive functions) in youth water polo players according to their playing position. Additionally, a one-way ANCOVA analysis with Bonferroni correction was used with the Stroop test outcomes as dependent variables, playing position as the independent variable (1 for a goalkeeper, 2 for a center-defender, 3 for a center-forward, 4 for a driver and 5 for a wing) and body mass and body height as covariates. Effect sizes (partial eta square) were reported [60], and the statistical significance level was set at *p* < 0.05.

## 3. Results

### 3.1. Baseline Characteristics of the Study Population

There were 106 players with a mean age of 14.14 ± 0.38 years (13–15 years) divided into five groups according to their playing position in the team: 15 goalkeepers (14.2%), 21 center-defenders (19.8%), 17 center-forwards (16.0%), 34 drivers (32.1%) and 19 wings (17.9%). The average body weight and height were 66.74 ± 11.21 kg and 176.65 ± 7.80 cm, respectively. Table 1 shows the data on the total sample (N = 106), reporting anthropometric characteristics, specific functional swimming capacities and cognitive functions.

### 3.2. One-Way ANOVA

We detected significant differences between different playing positions in anthropometric characteristics and specific functional swimming capacities, as shown in Table 2. Center-forwards had bigger body masses (77.99 ± 9.91 kg) and were taller (180.73 ± 5.98 cm) than drivers (63.18 ± 9.87 kg; 174.00 ± 6.34 cm) and wings (60.37 ± 8.16 kg; 171.83 ± 6.24 cm) (Table 2). Also, they were slower than drivers and wings in the 50 m crawl (center-forwards 31.27 ± 1.68 s vs. drivers 29.74 ± 1.28 s and wings 29.58 ± 2.09 s, *p* = 0.016) and the 400 m crawl (center-forwards 323.18 ± 14.67 s vs. drivers 311.53 ± 11.98 s, *p* = 0.020), as shown in Table 2. We found that players also differed in the results of the Stroop test. The wings performed faster than center-forwards in the StroopOff time (wings 57.14 ± 10.04 s vs. center-forwards 67.03 ± 9.72 s, *p* = 0.016), as well as in the StroopOn time (wings 66.18 ± 15.86 s vs. center-forwards 80.24 ± 15.64 s, *p* = 0.019), respectively.

### 3.3. ANCOVA

Table 3 reports differences in the cognitive functions of youth water polo players in different playing positions assessed with the Stroop test when controlled for covariates, body mass and body height. The ANCOVA analysis revealed no significant differences, with the exception of one significant difference in the StroopOn minus StroopOff time, where the ANCOVA model was significant.

The partial eta squared as a measure of the effect size showed a medium effect for all Stroop test variables (StroopOff η^2^ = 0.102, StroopOn η^2^ = 0.127; StroopOff + StroopOn η^2^ = 0.115, and StroopOn minus StroopOff η^2^ = 0.160). This indicates that, when controlling for the effect of body mass and body height, the different playing positions differ in their executive function performance, with a moderate effect size.

## 4. Discussion

To the best of our knowledge, this is the first study to report the association of cognitive functions and playing positions in youth water polo players. The aim of this study was to evaluate the level of selected cognitive features in youth water polo players while considering their assigned position on the field, as well as their anthropometric characteristics and specific swimming capacities. The results of this study indicated significant differences in psychomotor ability, response inhibition and motor speed between players in different playing positions, showing that wings performed faster in all measures of the Stroop test than players in the center-forward position. Additionally, center-forwards had a bigger body mass index than other playing positions, and they were slower in the 50 m crawl and the 400 m crawl than perimeter players and wings. This result can be explained by the fact that the playing position of wings requires more dynamism, explosiveness and agility than center-forwards. Unlike wings, center-forwards have to fight for the position with the defenders through constant physical contact and create a throw-in for themselves, which requires superior anthropometric characteristics [37,49]. To accomplish such a demanding role during the game, the center-forward is expected to have a wide range of external attention, the ability of multi-switching and a high level of working memory.

The importance of cognitive functions in sports has been widely demonstrated in previous studies [21,61,62,63]. Formenti et al. showed that the combination of cognitive functions (executive control and perceptual speed) and volleyball-specific skills was found to be useful for discriminating players of different competitive levels [61], similar as in soccer [63] or handball [2,12]. Another importance of cognitive functions in numerous sports shown in the previous literature (soccer, handball, volleyball, water polo, tennis and karate) mostly presented differences in cognitive functions and sport-specific physical performance for discriminating players of different competitive levels [2,3,8,13,20,43,64,65,66,67] and for talent identification [20,43,64,68]. Kalen et al. [3] reported that higher-skilled athletes performed better on cognitive function tests than lower-skilled athlete. Similar results were obtained by Krawczyk et al. [69] regarding handball goalkeepers playing in the champions league and super league when comparing simple and choice reaction times.

Recent studies highlight the need for a position-specific approach to the study of water polo, but the literature about the importance of cognitive functions in water polo is scarce [30,37,38,46,53,55]. Figuratively, water polo could be described as a combination of handball and swimming [37]; therefore, our results, which consider cognitive functions in different playing positions, can be partially compared with some previous research in handball. Blecharz et al. [2] presented that goalkeepers showed the shortest reaction time in reading words (neutral text color), with the highest tendency to read interference (difference between reading a neutral text and a colored text), indicating their high reactivity to visual stimuli. Additionally, Kiss and Balogh [12] found that goalkeepers, wingers, and playmakers had faster reaction times compared to pivot and back players, similar as in our study, which presented that center-forwards showed the slowest psychomotor speed. They also observed that goalkeepers committed fewer errors than pivot and back players when performing the task quickly. Therefore, it can be hypothesized that a smaller range of stimuli that a goalkeeper must process facilitates a quicker decision on what to do next and how to do so quickly and effectively [12]. In our study, wings showed the shortest reaction time and psychomotor speed, while goalkeepers followed them in comparison to center-forwards. These results are congruent with Silva’s approach in handball, suggesting that there is a similarity of cognitive demands for players assigned to different positions [27,70]. The exception comprises goalkeepers whose role on the court is different compared to the other players [27,38,44,70].

Trecroci et al. [21] suggested that volleyball players with superior basic cognitive functions (expressed by cumulated cognitive score) presented better sport-specific physical performance (expressed by the cumulated motor score). This finding is in line with a recent study that investigated the relationship between cognitive functions and sport-specific motor skills in young soccer players [13]. In that study, the cumulated score of cognitive tests (measuring attention window, perceptual load, multiple object tracking and working memory) was found to be associated with the cumulated score of motor tests [13]. Specifically, attention window and working memory were positively correlated with dribbling, ball control and ball juggling [13]. Based on those results, it could be speculated that well-developed cognitive functions may contribute to enhance players’ sport-specific skills within a game in unpredictable situations. In water polo, players play in water, an unnatural and complex environment, where they have to pay attention to the ball, teammates, and opponents’ movements. This dynamic environment stimulates their cognitive demands, encouraging players to find the best solution with the increasing complexity of the game [21,71,72]. Scharfen et al. [13] showed that the diagonal attention window (AW) was positively correlated with dribbling performance. This may suggest that athletes who have a wider AW also have advanced dribbling skills, which may enable them to execute early reactions in their sensorimotor system to make their performance more efficient. For example, in a game situation where the athlete is dribbling and simultaneously keeping an eye on the ball, his teammates and his opponents, helping him avoid contact with opponents and be more efficient in dribbling is important. Such activities also happen in water polo, which is in a different environment, namely water, and a broader AW could be beneficial for water polo players to spot their teammates easier and pass them the ball. Furthermore, Kiss and Balogh [12] demonstrated that handball players in pivot positions were outperformed by players in other positions in reaction when selecting adequate figures. Additionally, playmakers reacted with faster reaction time and decision-making than players in other positions. Pivots reacted slower, but that is in line with their role in the game, while they have fewer stimuli and less incorrect answers. Playing positions in water polo can be directly compared to handball, where center-forwards play a similar role as pivots; therefore, our results are in line with Kiss and Balogh’s in handball.

The positive association between cognitive and sport-specific performance domains is in line with the large body of the literature [3,5], which reports a substantial relationship between general motor and cognitive skills in children [9,73], adolescents [13,21,48,74] and adults [2,75].

Adding cognitive tests to assess players’ performance is supported by many previous studies, reporting a high association and overlap between cognitive functions and important aspects of sports performance, such as game intelligence, which is crucial for success in high-performance sports and still hardly measurable [8,76,77]. Therefore, it would be advantageous to include more participants in each playing position in future studies, which could help to clarify the reported model and possibly explain in which playing positions are cognitive differences consistently different. Such results may provide coaches with a more extensive picture of the players’ profiles in a multidimensional way and may help to assign them to the specific playing position within the team [2,13,63].

It is important to highlight that cognitive functions in athletes can be improved with cognitive training. Therefore, our results can be helpful for discriminating players for different playing positions and preparing cognitive training for each of them. Cognitive training aiming at improving athletic performance should be performed in a situation closely related to tasks performed on the field. Otherwise, skill transfer will be limited [61,62,78,79,80].

In the present work, we also analyzed the anthropometric characteristics and specific functional swimming capacities of youth water polo players playing in different playing positions, showing that center-forwards had bigger body weight and height than drivers and wings. Also, they were significantly slower in the 50 m crawl and the 400 m crawl than drivers and wings, which is in accordance with the demands of their playing position during the game. The center-forward position implements demanding technical and tactical elements, requiring more strength and power, as well as highly consistent athletic performance, than drivers or wings, who need to be faster and show better agility in the water.

In a former study, Kondrič et al. [37] examined the anthropometric and body composition characteristics and physical fitness of 110 junior water polo players with different playing positions. Based on their results, center-forwards had significantly higher body weight, BMIs, and larger subscapular skinfolds compared to other players. Concerning the physical condition measured by swimming tests, they found that the drivers achieved the best test results in 25 m and 400 m sprints, but without significant differences [37]. However, swimming tests are not suitable to describe the performance status of players during the training sessions and games, which both consist of technical and tactical components requiring further skills as well. Unfortunately, the accurate monitoring of players during training and games is still unresolved, making it difficult to collect performance data [37]. Therefore, analyzing cognitive functions, mental strength and game intelligence may contribute to developing adequate training programs for each playing position in water polo.

As water polo experienced recent rule changes [35,81], in order to make the game more attractive, actions during the game became faster, while the reaction time and decision-making time for players in specific playing positions became shorter. It could be speculated that a shorter water polo field of play will require different roles for players. For example, goalkeepers will participate more during the transition and defense actions, requiring well-developed anticipation and a fast reaction time. Players who can reach the 2 m-line first will play the center-forward position, which could change the definition and requirements for that specific position. Therefore, implementing cognitive training based on the demands of each playing position would be advantageous in the improvement of the training process in water polo.

### Limitations

The main limitation of this study is that it was conducted in laboratory settings, although the tasks players performed involved the functions needed when playing on the court. It would be advantageous to use sport-specific stimuli as suggested by Kalen et al. [3] who found that tests using sport-specific stimuli were considerably more successful in differentiating higher- and lower-skilled athletes than tests with non-sport-specific stimuli. However, it still remains unclear which cognitive tests should be used for these purposes. Also, in the present study, the number of athletes stratified by playing positions is limited, and we tested only male water polo players. Cognitive functions are influenced by many variables, such as age [82,83] and sex [84,85]. For these reasons, any form of generalization should be avoided, and results should be interpreted with caution. Therefore, we suggest using a consistent sample per playing position, increasing the number of participants, analyzing female water polo players as well and, if possible, conducting a longitudinal data analysis, in future studies.

## 5. Conclusions

In conclusion, this study demonstrated significant differences between different playing positions in youth water polo players, specifically between center-forwards and wings. They performed faster than center-forwards in all tested variables of the Stroop test, measures of psychomotor ability, response inhibition and motor speed, as well as in specific swimming capacities measured in the 50 m crawl and the 400 m crawl. On the contrary, center-forwards had a bigger body mass index than players in other playing positions, with the exception of center-defenders. However, caution should be used when interpreting these findings because differences in cognitive functions were not significant when controlling for body mass and body height. Thus, more studies with more participants should be conducted to confirm our results and possibly explain in which playing positions are cognitive differences consistently different.

Considering that cognitive functions may contribute to the development of game intelligence and play important role in successful long-term athlete development, this study can be an important basis for establishing developmental recommendations for players with distinct playing positions to improve their performance, taking into account their cognitive functions, anthropometric characteristics and specific functional swimming capacities. Additionally, this knowledge could be used by coaches to test athletes not only physically but also on the cognitive domain. Developing novel individualized training strategies and adding mentally demanding technical and tactical elements to their everyday training might advance players’ athletic development and ensure successful field efficiency.

## Figures and Tables

**Table 1:** Anthropometric characteristics and performance tests for all tested players (N = 106).

**Anthropometric Characteristics** (N = 106)		
	**Mean ± SD**	**95% CI**
Age (years)	14.15 ± 0.36	14.082–14.219
Body height (cm)	176.65 ± 7.80	175.024–178.273
Body mass (kg)	66.74 ± 11.21	64.407–69.077
Body mass index (kg/m^2^)	21.30 ± 2.78	20.724–21.882
**Specific swimming capacities**		
Crawl, 25 m (s) N = 91	13.70 ± 0.74	13.533–13.871
Crawl, 50 m (s) N = 91	30.10 ± 1.81	29.722–30.477
Crawl, 100 m (s) N = 91	96.41 ± 32.23	84.589–108.233
Crawl, 400 m (s) N = 91	314.62 ± 13.12	311.804–317.431
Breaststroke, 100 m (s) N = 15	96.90 ± 3.55	93.616–100.179
Butterfly, 100 m (s) N = 15	93.41 ± 10.84	83.390–103.433
Dribbling, 25 m (s) N = 91	14.65 ± 0.93	14.453–14.842
**Cognitive performance via the Stroop test** (N = 106)		
StroopOff time (s)	61.40 ± 9.40	59.589–63.210
StroopOn time (s)	72.52 ± 14.97	69.634–75.399
StroopOff + StroopOn time (s)	133.92 ± 23.66	129.358–138.473
Ontime minus Offtime (s)	11.12 ± 8.06	9.566–12.669
Incorrect runs total time (StroopOff) (s)	4.27 ± 6.39	3.042–5.504
Incorrect runs total time (StroopOn) (s)	6.307 ± 8.979	4.577–8.036
Successful times × attempts (Off) (s)	306.99 ± 47.01	297.94–316.05
Successful times × attempts (On) (s)	362.58 ± 74.83	348.17–376.99

Data are presented as the mean ± standard deviation and 95% CI. CI—confident interval.

**Table 2:** Comparison between playing positions.

Playing Positions	Goalkeepers N = 15	Center-Defenders N = 21	Center-Forwards N = 17	Drivers N = 34	Wings N = 19	*p* *
**Anthropometric characteristics** (N = 106)						
Age (years)	14.20 ± 0.41	14.14 ± 0.36	14.06 ± 0.24	14.15 ± 0.36	14.21 ± 0.42	0.756
Body height (cm)	180.93 ± 10.32 ^d^	178.03 ± 6.64	180.73 ± 5.98 ^d^	174.00 ± 6.34	171.83 ± 6.24 ^a,b^	0.001 *
Body mass (kg)	66.82 ± 12.69	68.46 ± 8.29	77.99 ± 9.91 ^c,d^	63.18 ± 9.87 ^b^	60.37 ± 8.16 ^b^	<0.001 *
Body mass index (kg/m^2^)	20.24 ± 2.25 ^b^	21.55 ± 1.92	23.92 ± 3.31 ^a,c,d^	20.81 ± 2.60 ^b^	20.42 ± 2.44 ^b^	0.001 *
**Specific swimming capacities**						
Crawl, 25 m (s) N = 91		13.64 ± 0.99	14.04 ± 0.69	13.69 ± 0.58	13.44 ± 0.70	0.160
Crawl, 50 m (s) N = 91		30.20 ± 2.06	31.27 ± 1.68 ^c,d^	29.74 ± 1.28 ^b^	29.58 ± 2.09 ^b^	0.016 *
Crawl, 100 m (s) N = 91		66.92 ± 4.29	69.05 ± 2.80	66.89 ± 2.83	66.13 ± 3.09	0.104
Crawl, 400 m (s) N = 175		312.25 ± 13.12	323.18 ± 14.67 ^c^	311.53 ± 11.98 ^b^	314.32 ± 10.84	0.020 *
Breaststroke, 100 m (s) N = 15	96.90 ± 3.55					NA
Butterfly, 100 m (s) N = 15	93.41 ± 10.84					NA
Dribbling, 25 m (s) N = 91		14.88 ± 1.13	14.76 ± 0.98	14.66 ± 0.74	14.28 ± 0.93	0.219
**Cognitive performance via the Stroop test** (N = 106)						
StroopOff time (s)	58.44 ± 6.99	62.68 ± 10.08	67.03 ± 9.72 ^d^	61.48 ± 8.15	57.14 ± 10.04 ^b^	0.016 *
StroopOn time (s)	66.81 ± 10.34 ^b^	71.50 ± 12.29	80.24 ± 15.64 ^a,d^	75.34 ± 15.57	66.18 ± 15.86 ^b^	0.019 *
StroopOff + StroopOn time (s)	125.26 ± 16.88	134.18 ± 21.80	147.27 ± 24.3 ^d^	136.81 ± 23.14	123.32 ± 25.38 ^b^	0.017 *
Ontime minus Offtime (s)	8.37 ± 5.13 ^b^	8.83 ± 5.47	13.21 ± 9.35 ^a,d^	13.86 ± 9.08	9.03 ± 7.79 ^b^	0.041 *
Incorrect runs total time (StroopOff) (s)	6.56 ± 8.43	4.97 ± 6.25	2.17 ± 6.90	5.05 ± 6.12	2.17 ± 3.72	0.159
Incorrect runs total time (StroopOn) (s)	5.46 ± 11.71	3.42 ± 6.09	6.68 ± 9.14	9.51 ± 9.46	4.09 ± 7.01	0.095
Successful times × attempts (Off) (s)	292.22 ± 34.93	313.38 ± 50.42	335.15 ± 48.59 ^d^	307.38 ± 40.75	285.71 ± 50.18 ^b^	0.016 *
Successful times × attempts (On) (s)	334.07 ± 51.69	357.52 ± 61.44	401.21 ± 78.22 ^d^	376.68 ± 77.86	330.89 ± 79.30 ^b^	0.019 *

Data are presented as the mean ± standard deviation. * ANOVA with the post hoc Scheffé test for all pairwise comparisons; *p* < 0.05. ^a^ comparison with goalkeepers (*p* < 0.05). ^b^ comparison with center-attackers (*p* < 0.05). ^c^ comparison with perimeter players (*p* < 0.05). ^d^ comparison with wings (*p* < 0.05).

**Table 3:** ANCOVA analysis showing differences in cognitive functions between different playing positions.

		Mean	SE	95% CI	F	*p*
**StroopOff time**	**R^2^ = 0.102 R^2^ adjusted = 0.038**				1.587	0.161
	Goalkeeper (N= 15)	58.61	2.32	54.010–63.202		
	Center-backward (N = 21)	62.68	1.95	58.800–66.554		
	Center-forward (N = 17)	66.63	2.20	62.263–70.994		
	Perimeter (N = 34)	61.24	1.55	58.165–64.314		
	Wings (N = 18)	58.59	2.19	54.229–62.944		
**StroopOn time**	**R^2^ = 0.127 R^2^ adjusted = 0.065**				2.035	0.070
	Goalkeeper (N = 15)	67.005	3.730	59.603–74.406		
	Center-backward (N = 21)	71.503	3.147	65.260–77.747		
	Center-forward (N = 17)	79.768	3.543	72.738–86.798		
	Perimeter (N = 34)	75.058	2.495	70.107–80.009		
	Wings (N = 18)	67.822	3.537	60.804–74.840		
**StroopOff + StroopOn time**	**R^2^ = 0.115 R^2^ adjusted = 0.065**				1.818	0.105
	Goalkeeper (N = 15)	125.610	5.864	113.976–137.245		
	Center-backward (N = 21)	134.180	4.946	124,366–143.995		
	Center-forward (N = 17)	146.396	5.570	135,345–157.448		
	Perimeter (N = 34)	136.298	3.923	128.514–144.081		
	Wings (N = 18)	126.408	5.560	115.376–137.441		
**StroopOn minus StroopOff time**	**R^2^ = 0.160 R^2^ adjusted = 0.100**				2.674	0.020
	Goalkeeper (N = 15)	8.399	2.043	4.346–12.452		
	Center-backward (N = 21)	8.827	1.723	5.408–12.246		
	Center-forward (N = 17)	13.139	1.940	9.290–16.990		
	Perimeter (N = 34)	13.818	1.367	11.107–16.530		
	Wings (N = 18)	9.236	1.937	5.393–13.079		

ANCOVA, including body mass and body height as covariates in the model comparing the cognitive functions of youth water polo players in different playing position; Bonferroni correction; *p* < 0.05.

## Data Availability

The data presented in this study are available upon request from the corresponding author.
